# Opioid prescribing among new users for non-cancer pain in the USA, Canada, UK, and Taiwan: A population-based cohort study

**DOI:** 10.1371/journal.pmed.1003829

**Published:** 2021-11-01

**Authors:** Meghna Jani, Nadyne Girard, David W. Bates, David L. Buckeridge, Therese Sheppard, Jack Li, Usman Iqbal, Shelly Vik, Colin Weaver, Judy Seidel, William G. Dixon, Robyn Tamblyn

**Affiliations:** 1 Centre for Epidemiology Versus Arthritis, Centre for Musculoskeletal Research, University of Manchester, Manchester, United Kingdom; 2 Department of Rheumatology, Salford Royal Foundation Trust, Salford, United Kingdom; 3 Department of Epidemiology, Biostatistics & Occupational Health, University of McGill, Montreal, Canada; 4 Clinical and Health Informatics Research Group, McGill University, Montreal, Canada; 5 Harvard Medical School, Boston, Massachusetts, United States of America; 6 Division of General Internal Medicine, Brigham and Women’s Hospital, Boston, Massachusetts, United States of America; 7 Department of Health Policy and Management, Harvard T.H. Chan School of Public Health, Boston, Massachusetts, United States of America; 8 International Centre for Health Information Technology (ICHIT), Taipei Medical University, Taipei City, Taiwan; 9 Graduate Institute of Biomedical Informatics, College of Medicine Science and Technology, Taipei Medical University, Taipei City, Taiwan; 10 Applied Research and Evaluation Services, Alberta Health Services, Calgary, Canada; 11 Department of Community Health Science, Cumming School of Medicine, University of Calgary, Calgary, Canada; Addis Ababa University / King’s College London, ETHIOPIA

## Abstract

**Background:**

The opioid epidemic in North America has been driven by an increase in the use and potency of prescription opioids, with ensuing excessive opioid-related deaths. Internationally, there are lower rates of opioid-related mortality, possibly because of differences in prescribing and health system policies. Our aim was to compare opioid prescribing rates in patients without cancer, across 5 centers in 4 countries. In addition, we evaluated differences in the type, strength, and starting dose of medication and whether these characteristics changed over time.

**Methods and findings:**

We conducted a retrospective multicenter cohort study of adults who are new users of opioids without prior cancer. Electronic health records and administrative health records from Boston (United States), Quebec and Alberta (Canada), United Kingdom, and Taiwan were used to identify patients between 2006 and 2015. Standard dosages in morphine milligram equivalents (MMEs) were calculated according to The Centers for Disease Control and Prevention. Age- and sex-standardized opioid prescribing rates were calculated for each jurisdiction. Of the 2,542,890 patients included, 44,690 were from Boston (US), 1,420,136 Alberta, 26,871 Quebec (Canada), 1,012,939 UK, and 38,254 Taiwan. The highest standardized opioid prescribing rates in 2014 were observed in Alberta at 66/1,000 persons compared to 52, 51, and 18/1,000 in the UK, US, and Quebec, respectively. The median MME/day (IQR) at initiation was highest in Boston at 38 (20 to 45); followed by Quebec, 27 (18 to 43); Alberta, 23 (9 to 38); UK, 12 (7 to 20); and Taiwan, 8 (4 to 11). Oxycodone was the first prescribed opioid in 65% of patients in the US cohort compared to 14% in Quebec, 4% in Alberta, 0.1% in the UK, and none in Taiwan. One of the limitations was that data were not available from all centers for the entirety of the 10-year period.

**Conclusions:**

In this study, we observed substantial differences in opioid prescribing practices for non-cancer pain between jurisdictions. The preference to start patients on higher MME/day and more potent opioids in North America may be a contributing cause to the opioid epidemic.

## Abstract 2

Using population-based cohorts in North America, UK and Taiwan, Dr. Meghna Jani and colleagues describe opioid prescribing rates in patients with non-cancer pain who are using opioids for the first time.

## Author summary

**Why was this study done?:**

- An opioid crisis has affected several high-income countries, especially in North America, followed by sharp increase in opioid-related deaths in recent years.
- There is little evidence on how opioid prescribing rates compare internationally and the differences between countries in the type, strength, starting dose of opioids, and whether these characteristics change over time.

**What did the researchers do and find?:**

- We analyzed data from 2,542,890 adults who were prescribed opioids for the first time between 2006 to 2016 in 4 countries and 5 jurisdictions (United States, Boston; Canada, Quebec and Alberta; United Kingdom; Taiwan).
- The highest opioid prescribing rates in 2014 were observed in Alberta at 66/1,000 persons compared to 52, 51, and 18/1,000 in the UK, US, and Quebec, respectively, after standardizing for age and sex.
- Median morphine milligram equivalents (MMEs)/day at first use was highest in Boston at 38 MME/day and in contrast were less than half of the US in the UK (12 MME/day) and Taiwan (8 MME/day).
- Oxycodone was the first prescribed opioid in 65% of patients of the US cohort compared to 14%, Quebec 4%, Alberta; 0.1%, UK; and none in Taiwan.
- At initiation of an opioid, 20% of US patients, 13% of Quebec patients, and 10% of Albertans exceeded the US CDC–recommended threshold of 50 MME/day compared to 0.6% in the UK and 0.2% in Taiwan.

**What do these findings mean?:**

- In one of the first international comparisons to our knowledge, the preference to initiate patients on higher MME/day and more potent opioids in North America may be a contributing factor to the opioid epidemic.

## Introduction

Over the last 15 years, an opioid crisis has gripped several countries accompanied by a parallel increase in opioid prescribing rates, opioid-related morbidity, and mortality. In 2017, The Global Burden of Diseases, Injuries, and Risk Factors Study estimated that 40.5 million people were opioid dependent, and 109,500 people died from an opioid overdose [1]. The United States, in particular, has been affected by a crippling opioid epidemic, with currently over 130 deaths per day due to opioid overdoses, including unintentional deaths [2]. While several high-income countries report increases in opioid-related deaths and dependence, marked differences exist between regions.

The US and Canada have the highest estimated prevalence of age-standardized opioid overdose deaths worldwide, as well as the highest per capita opioid consumption [3]. Between 1999 and 2017, an astonishing 399,233 Americans died of an opioid overdose [4], with Canada facing a similar stark predicament particularly in the western provinces of Alberta and British Columbia [5]. Additionally, following the Coronavirus Disease 2019 (COVID-19) pandemic, jurisdictions such as British Columbia are now seeing the highest opioid overdose frequencies ever observed [6]. While such overdoses may be in part secondary to illicit drug use, the majority of people who use heroin started with prescription opioids [7]. In European countries such as the United Kingdom and the Netherlands, opioid-related mortality has risen albeit to a much lesser extent [8,9]. Differences in prescribing practices may be at the root of this epidemic.

Few studies have investigated opioid prescribing differences internationally. A previous study of dentists found that the rate of opioid prescribing was 37 times higher in the US than in England [10]. An additional challenge when performing international comparisons is that each country and jurisdiction may have differences in prescription rules, incentives for funding specific opioids over others, availability of different opioid drugs, and differences in training of healthcare professionals. However, it is possible that country-specific differences in the type and strength of opioid initiated, and patient populations being prescribed opioids, may be fueling the epidemic.

Harnessing the strengths of the International Pharmacovigilance Network [11], we used electronic health records (EHRs) and population-based administrative data to compare opioid prescribing rates in patients without cancer across 5 centers in 4 countries: Canada (Quebec and Alberta), US (Boston), UK, and Taiwan. We evaluated differences in the type, strength, and starting dose of medication and whether these characteristics changed over time in people who use opioids for the first time.

## Methods

### Study design and population

We assembled new opioid user cohorts in 5 jurisdictions and 4 countries (Canada, US, UK, and Taiwan) using data from January 1, 2006 to January 1, 2016. To be included in the cohort, patients had to be ≥18 years of age and have at least 1 prescribed or dispensed opioid (for simplicity, we will use the term “prescribed” for the remainder of the text). Patients were excluded (i) if they had an ICD-10 code (or Read Code in the UK database) for malignancy with the exception of those with non-melanoma skin cancer or (ii) if there was a history of opioid use in the 2 years prior to first prescription. Details on how cohorts were derived and additional cohort descriptions are outlined in the S1 Appendix. This study is reported as per the Strengthening the Reporting of Observational Studies in Epidemiology (STROBE) guideline (S1 STROBE Checklist).

### Data sources and context

#### Canada: Quebec

Data were retrieved from the Montreal Population Health Record between 2006 and 2014. This was a population-based 25% random sample of the 4.1 million residents of Montreal, Quebec, which is dynamically updated each year to account for in- and out-migration.

#### Canada: Alberta

Alberta has a publicly funded health system that provides coverage to approximately 4.4 million residents. Data were extracted from the Alberta Health Services repository for all Albertans who were continuously registered for coverage with the Alberta Health Care Insurance Plan in the 2 years prior to the index prescription for the period 2009 to 2015.

#### United States: Boston, Massachusetts

Data were retrieved from the Partners HealthCare Research Patient Data Registry between 2010 and 2015, which provides healthcare for 3 of the 5 million residents in Boston and its surrounding areas.

#### United Kingdom

The Clinical Practice Research Datalink (CPRD) Gold, a deidentified EHR database for primary care, was used to retrieve all eligible patients between 2006 and 2015, representative of the national population.

#### Taiwan

The Taiwanese National Health Insurance (NHI) system database was used, which provides national health coverage to the majority of the Taiwanese population (99%) of 23 million patients. Data from NHI were available between 2009 and 2012.

### Opioid prescribing and daily dose

The Anatomic Therapeutic Classification system was used to map national drug names and identification numbers to a common nomenclature based on opioids that were prescribed during the study period. To allow direct comparison of doses and opioid potencies across different formulations, we calculated the morphine milligram equivalents (MMEs)/day for each patient. MME/day was defined as the daily dose for each prescription multiplied by the equivalent analgesic ratio of the opioid type as specified by the US Centers for Disease Control and Prevention (CDC) [12]. For instance, a patient on oxycodone 40 mg once a day would equate to 60 MME/day (40 × 1.5). If patients were started on more than 1 opioid, we categorized them into a separate “combination drug” group where the MME/day for each opioid were added together. In Taiwan, opioids are only available by prescription. Therefore, to make the data sources comparable between the countries, we excluded low-dose opioid products (mainly codeine) that are available as over- or behind-the-counter drugs in other jurisdictions from the analysis.

### Patient characteristics

#### Age and sex

Date of birth and sex were retrieved from the administrative data in Canada and Taiwan as these data are verified at the time of enrollment in the health plan. For the US and UK, these data were retrieved from EHRs. To protect confidentiality, age was grouped into 7 categories (18 to 24; 25 to 34; 35 to 44; 45 to 54; 55 to 64; 65 to 74; ≥75 years).

#### Comorbidities

We used standard diagnostic codes (ICD9, ICD10, and Read Codes) retrieved from EHRs, medical services claims, and hospitalizations in the 2 years prior to the first opioid prescription to measure clinically relevant comorbidities. The Charlson comorbidity index, the weighted sum of predefined conditions that increase the risk of mortality, was used to measure disease complexity/severity [13]. Chronic non-cancer pain conditions were identified using a validated ICD9 and ICD10 code set mapped to Read Codes and included lumbar pain, neck and back problems, fibromyalgia, regional pain syndromes, painful neuropathic disorders, pain disorders with psychosocial dysfunction, and unclassified chronic pain problems [14]. Depression included mild, moderate, major single, or recurrent depressive disorder with or without psychotic symptoms, adjustment reaction, and mixed anxiety and depression. Substance abuse included alcohol abuse and illicit drug use.

#### Concurrent drug use

This was defined as an active prescription or dispensed supply on the date of the first opioid prescription (index date), which may be prescribed for pain (antidepressants, gabapentanoids) and/or increase the risk of harm with opioids (benzodiazepines, antipsychotics). Our definition of benzodiazepines excluded non-benzodiazepine gamma-aminobutyric acid agonists or “Z-drugs” such as a zolpidem, zopiclone, and zaleplon.

### Statistical analysis

Descriptive statistics were used to compare the drug utilization of opioids in each country. In each jurisdiction, age- and sex-standardized rates of opioid prescribing were calculated using the direct standardization method using the UK age–sex population distribution [15] as the reference population. For each study year and for each cohort, we calculated the age-and sex-stratified opioid prescribing rates. The denominators used for each cohort represent the underlying population from which the cohort was drawn. To obtain the expected numbers of people who use opioids, we multiplied the age–sex rates of each jurisdictional population by the n in each age–sex stratum of the UK reference population. These expected numbers of people who use opioids were added for each cohort and then divided by the reference population to obtain the standardized rate for the respective jurisdiction. For each jurisdictional cohort, the number of persons alive at the start of each year of follow-up was the denominator, and the number of persons newly prescribed an opioid in that year was the numerator. Although a prospective analysis plan has not been included, the objectives and methods of this work were determined at the outset of the planned study according to the unmet needs in the literature and were not adapted subsequently. No data-driven changes to the analysis plan were undertaken after obtaining the data.

Statistical analyses were performed using SAS version 9.4. The study was approved by the McGill institutional review board, Montreal (IRB Study number A01-E03-13B); University of Calgary ethics (REB18-2014_REN1), Alberta; Partners Human Research Committee (Protocol 2011P000773) Boston; the Independent Scientific Advisory Committee of CPRD (Protocol 16_278) for UK data; and the Taipei Medical University Joint Institutional Review Board, Taiwan (Ref: 076/20160306).

## Results

### Characteristics of people on opioids

We identified 2,542,890 patients who met the eligibility criteria across the 5 jurisdictions (Table 1). The highest proportions of people who use opioids (who were previously opioid naïve) in most cohorts were between the ages of 25 to 54 in the US, UK, Alberta, and Taiwanese cohorts. In the Quebec cohort, patients ≥75 comprised 23% of the total cohort (Table 1). Preexisting conditions associated with opioid use [12] varied considerably between cohorts. Depression was most common in the Canadian cohorts: Quebec 20% and Alberta 13%. Substance abuse prior to first opioid prescription ranged between 0.8% in Taiwan and 7% in the Canadian cohorts. Chronic pain conditions were most commonly reported in Taiwan (70%) and least frequently in Boston (22%) (Table 1). Benzodiazepines were most commonly concurrently prescribed with opioids in Quebec (20%), Taiwan (18%), Boston (7%), and UK (7%). Gabapentinoid use ranged between 7% in Quebec and 2% in Taiwan. Antidepressants were more commonly coprescribed with opioids in the Quebec (34%), Alberta (11%), and UK (7%) cohorts.

**Table 1:** The characteristics of people on opioids by jurisdiction.

	Canada		United States	United Kingdom	Taiwan
	Quebec	Alberta	Boston		Excl. low-dose codeine
Patient characteristic	*N* = 26,871	*N =* 1,420,136	*N* = 44,690	*N* = 1,012,939	*N* = 38,254
**Age at first opioid prescription or dispensation**					
18 and 24 years	878 (3.3%)	183,602 (12.9%)	2,324 (5.2%)	7,7305 (7.6%)	2,110 (5.5%)
25 and 34 years	2,359 (8.8%)	269,756 (19.0%)	7,781 (17.4%)	142,611 (14.1%)	4,433 (11.6%)
35 and 44 years	3,048 (11.3%)	262,824 (18.5%)	9,072 (20.3%)	174,624 (17.2%)	5,277 (13.8%)
45 and 54 years	4,228 (15.7%)	263,960 (18.6%)	9,371 (21.0%)	173,267 (17.1%)	7,337 (19.2%)
55 and 64 years	4,439 (16.5%)	221,175 (15.6%)	8,410 (18.8%)	162,455 (16.0%)	7,433 (19.4%)
65 and 74 years	5,787 (21.5%)	125,963 (8.9%)	4,930 (11.0%)	133,385 (13.2%)	5,805 (15.2%)
≥75 years	6,132 (22.8%)	92,856 (6.5%)	2,802 (6.3%)	149,292 (14.7%)	5,859 (15.3%)
**Sex**					
Female	18,406 (68.5%)	740,512 (52.1%)	30,121 (67.4%)	585,306 (57.8%)	21,290 (55.7%)
Male	8,465 (31.5%)	679,624 (47.9%)	14,569 (32.6%)	427,633 (42.2%)	16,964 (44.3%)
**Charlson comorbidity index**					
Very low score (0)	14,766 (55.0%)	1,143,973 (80.6%)	30,286 (67.8%)	653,939 (64.6%)	18,094 (47.3%)
Low score (1)	7,230 (26.9%)	191,054 (13.5%)	7,939 (17.8%)	206,422 (20.4%)	10,714 (28.0%)
Medium score (2–3)	3,677 (13.7%)	69,525 (4.9%)	4,427 (9.9%)	109,381 (10.8%)	7,557 (19.8%)
High score (≥4)	1,198 (4.5%)	15,584 (1.1%)	2,038 (4.6%)	43,197 (4.3%)	1,889 (4.9%)
**Comorbidity in 2 years before first opioid**					
Depression	5,404 (20.1%)	190,402 (13.4%)	3,895 (8.7%)	88,486 (8.7%)	801 (2.1%)
Substance abuse	1,807 (6.7%)	94,936 (6.7%)	1,370 (3.1%)	34,294 (3.4%)	290 (0.8%)
Pain	7,386 (27.5%)	483,310 (34.0%)	9,807 (21.9%)	298,662 (29.5%)	26,723 (69.9%)
**Concurrent drug use on the date first opioid**			
Gabapentinoids	1,975 (7.3%)	28,834 (2.0%)	1,417 (3.2%)	12,447 (1.2%)	755 (2.0%)
Benzodiazepines	5,360 (20.0%)	57,671 (4.1%)	3,211 (7.2%)	71,041 (7.0%)	6,980 (18.2%)
Antipsychotics	2,268 (8.4%)	25,060 (1.8%)	650 (1.4%)	22,479 (2.2%)	1,232 (3.2%)
Antidepressants	9,690 (36.1%)	152,173 (10.7%)	2,064 (4.6%)	72,622 (7.2%)	2,605 (6.8%)

### Rates of opioid use

**Fig 1:**
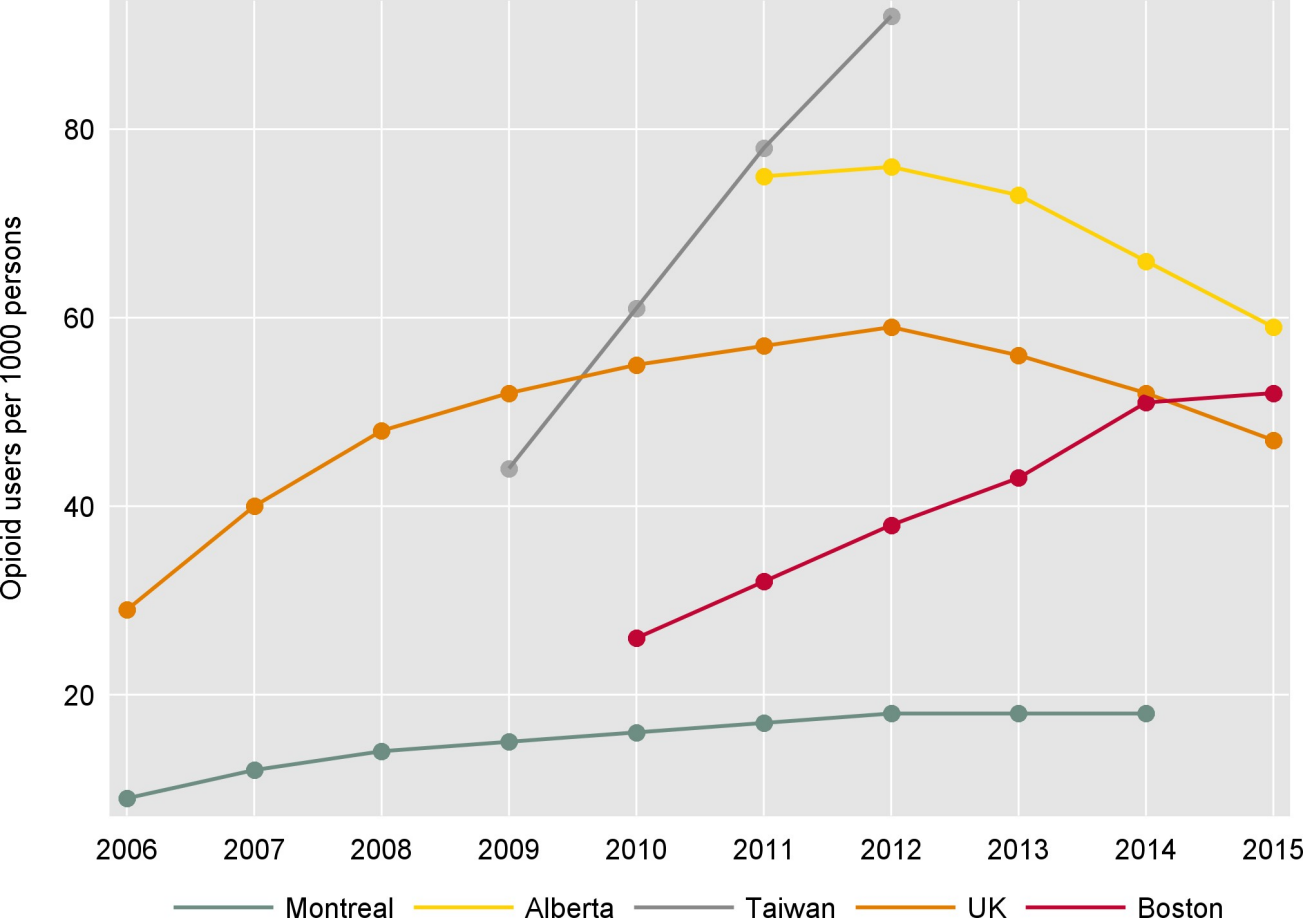
Age- and sex-standardized rates of opioid use per 1,000 population.

The highest age- and sex-standardized rate of opioid prescribing was observed in Taiwan at 92 per 1,000 persons in 2012, followed by Alberta at 66 per 1,000 persons (2014), and Boston at 52 per 1,000 persons (2015) (Fig 1). Prescribing rates peaked at different times, with the steepest rise observed in Taiwan and Boston. Taiwan rates more than doubled between 2009 and 2012 from 44 to 92 per 1,000 persons, Boston (26 to 52 per 1,000 persons, 2010 to 2015), UK (29 to 47 per 1,000 persons, 2006 to 2015), and Quebec (9 to 18 per 1,000 persons, 2006 to 2014). A decline in prescribing rates was observed in Alberta (75 to 59 per 1,000, 2011 to 2015). In Taiwan, the steep rise of prescribing coincided with an increase in reported pain diagnosis (15% in 2009; 18% in 2012) and a slight increase in Charlson comorbidity index (medium score increased from 4% to 5%) (S2 Appendix). In Boston, between 2010 and 2015, the proportion of people who used opioids who were ≥75 years increased from 5% to 8%, alongside an increase of pain diagnosis (20% to 27%) and the proportion of those with comorbidities, as represented by medium or high Charlson comorbidity score (S2 Appendix).

### Daily morphine milligram equivalents and type of opioid at initiation

**Fig 2:**
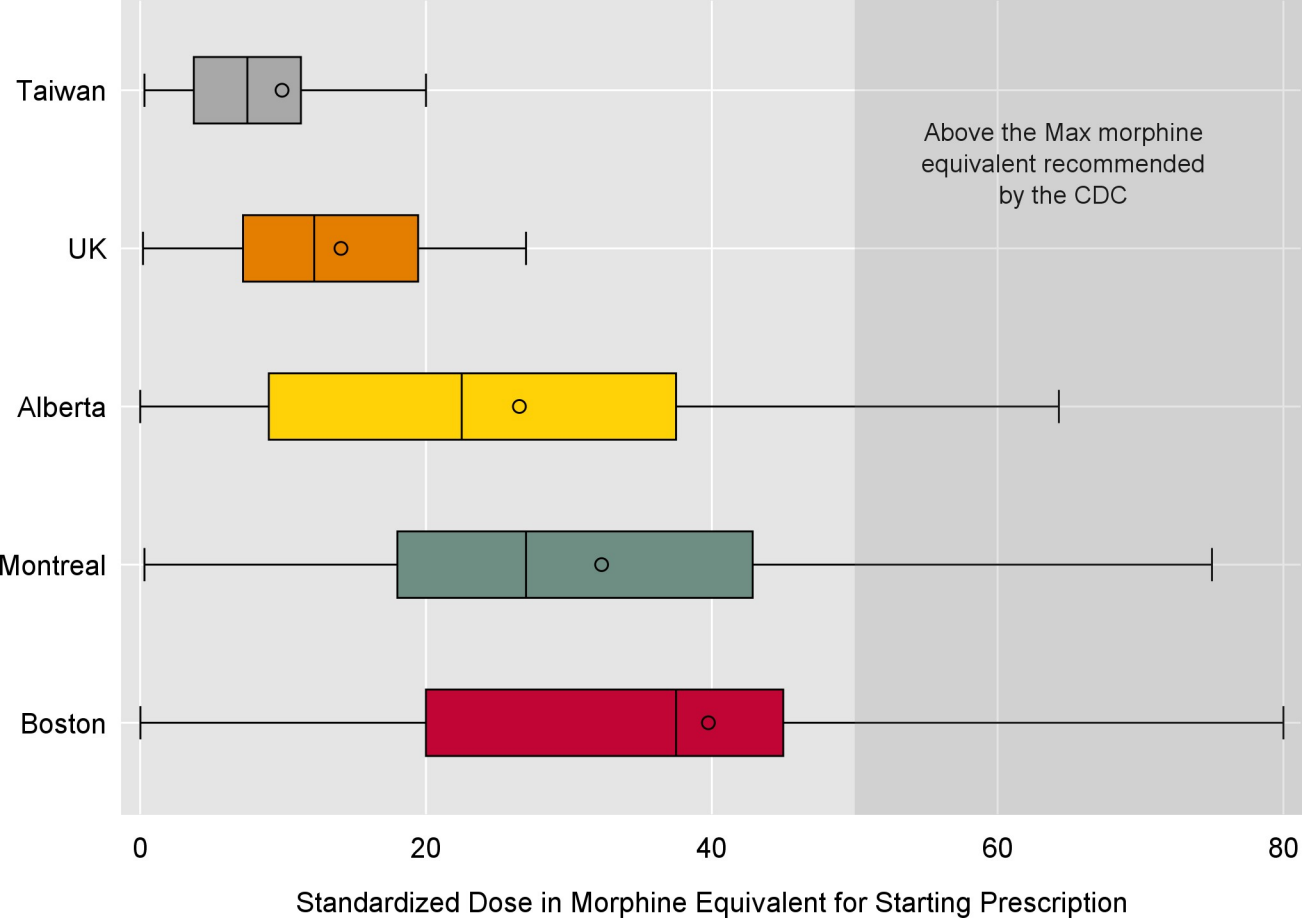
Daily dose of first opioid prescription in MMEs. The lower limit of the box plot represents the lowest standardized dose, the lower and upper ends of the colored box the interquartile range, the line in the box represents the median, and the circle the mean standardized dose. For this box plot, the upper limit of the box plot is set to the 95th percentile. CDC, Centers for Disease Control and Prevention; MME, morphine milligram equivalent.

The highest starting doses (median MME/day (IQR)) of opioids were in the North American jurisdictions—38 MME/day (20, 45) in Boston; 27 MME/day (18, 43) in Quebec; and 23 MME/day (9, 38) in Alberta (Fig 2). In contrast, starting doses were less than half of North American doses in the UK (12 MME/day (7, 20)) and Taiwan (8 MME/day (4, 11)). Moreover, 20.1% of Boston patients, 12.7% of Quebec patients, and 9.6% of Albertans exceeded the US CDC–recommended threshold of 50 MME/day at initiation compared to 0.6% in the UK and 0.2% in Taiwan.

**Fig 3:**
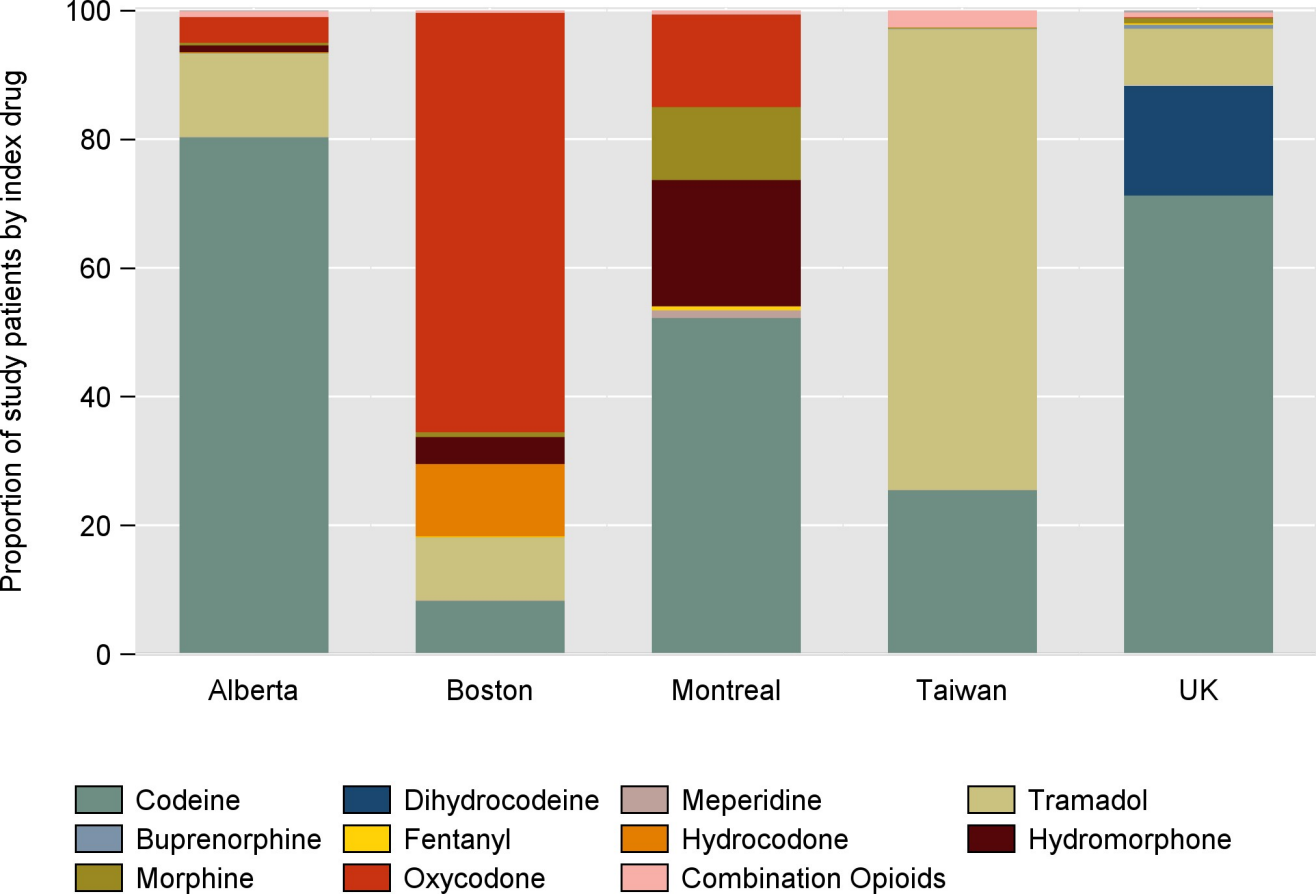
Type of opioid on initiation.

Differences in starting MME/day was primarily related to drug choice. Oxycodone was the most common initial opioid prescribed (65%) in Boston compared to 14% in Quebec, 4% in Alberta, and 0.1% in the UK and was not included in the Taiwanese drug formulary (Fig 3). In all jurisdictions besides Boston and Taiwan, codeine was the predominant opioid prescribed as first line therapy prescribed to 71% of people who use opioids in the UK, 80% in Alberta, and 52% in Quebec. Tramadol was the most prescribed first-line therapy in Taiwan (72%) and was frequently prescribed in Alberta (13%), Boston (10%), and the UK (9%). Hydrocodone was prescribed almost exclusively in the Boston cohort (11%), with hydromorphone being prescribed in 4% compared to 20% in the Quebec cohort. Morphine was prescribed first line most frequently in the Quebec cohort (11%), with <1% usage in the rest of the jurisdictions. Fentanyl was prescribed infrequently first line in Quebec (0.6%), UK (0.3%), and Boston (0.2%) and <0.01% of patients in Alberta and Taiwan.

### Opioid trends over time by country

**Fig 4:**
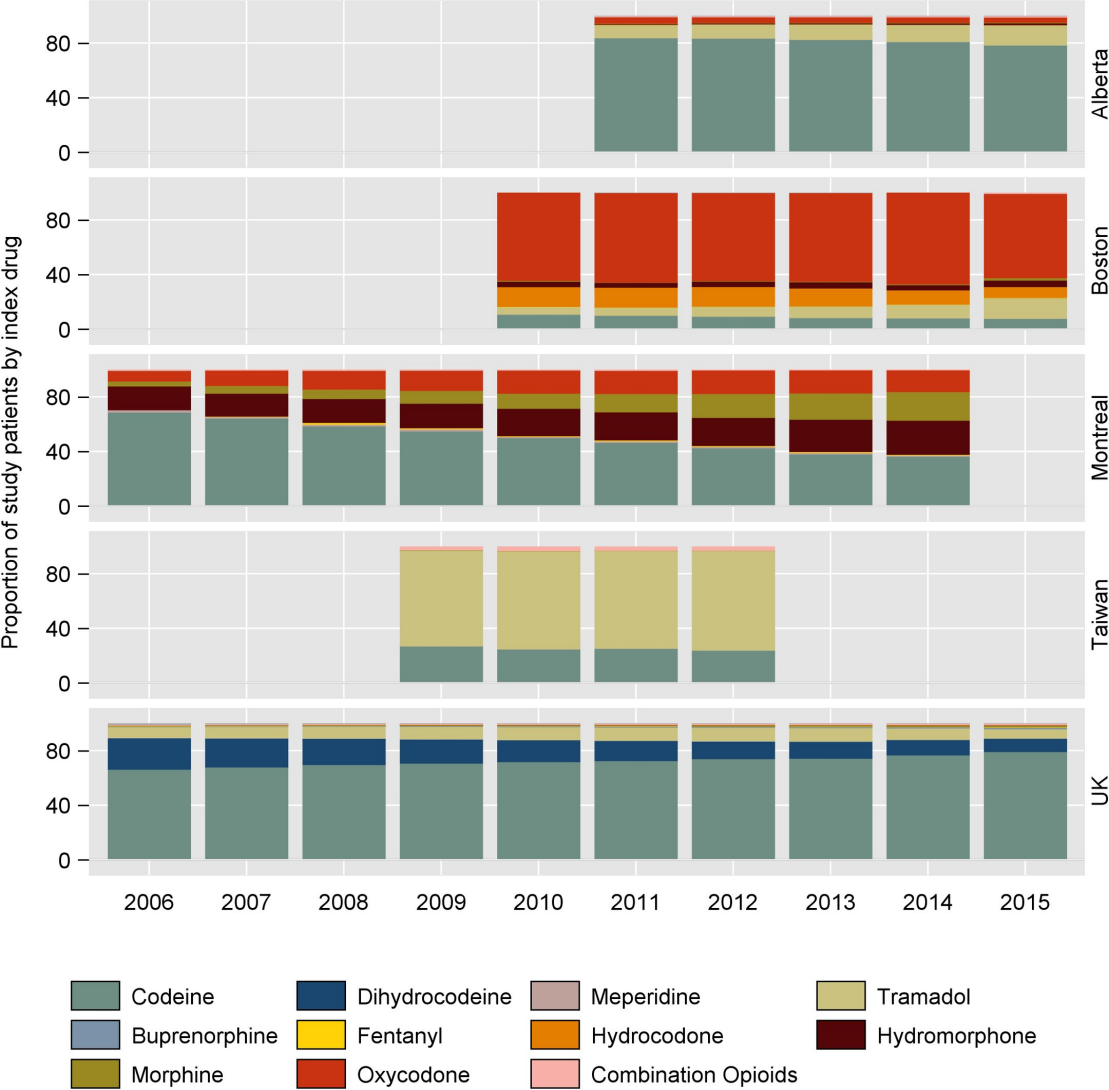
Opioid trends over time by drug.

Of interest, the proportion of patients started on codeine decreased over time in all cohorts, except the UK where the proportion started on codeine increased (Fig 4). The proportion of hydromorphone, oxycodone, and morphine increased steadily over time in the Quebec cohort. Tramadol prescribing increased during their observed time in Alberta (2011 to 2015), Boston (2010 to 2015), and Taiwan (2009 to 2012).

## Discussion

In a study of over 2.5 million patients over 5 international jurisdictions, differences in opioid prescribing were substantial. The median MME/day at initiation ranged from 38 MME/day in Boston (US) to 8 MME/day in Taiwan. Strikingly, the 95th percentile MME/day at initiation in the UK and Taiwan was lower than the median MME/day in the US. The highest standardized opioid prescribing rates in 2014 were observed in Alberta at 66/1,000 persons compared to 52, 51, and 18 per 1,000 persons in the UK, US, and Quebec, respectively. The most dramatic escalation of people who use opioids for the first time was observed in the Boston and Taiwanese cohorts during the observed timeframe.

Prescribed opioids are important drivers of the opioid epidemic [16–20]. High initial strength of opioids and daily dose have been linked to increased risk of opioid-related harms and long-term use [21,22]. In opioid-naïve patients, the risk of transitioning to long-term use begins as soon as the fifth day of exposure and increases with each incremental rise in cumulative dose highlighting the importance of initial opioid exposure [18]. Our study demonstrates that there are clear differences in both the type of opioid and MME/day prescribed at initiation in North America versus UK and Taiwan. The Boston cohort had a higher proportion of patients on higher potency opioids such as oxycodone, hydrocodone, and hydromorphone at initiation compared to Alberta, Taiwan, and the UK, which had a higher proportion of patients starting weak/moderate potency opioids such as codeine and tramadol. A similar observation was noted in a cross-sectional study comparing dental opioid prescriptions in the US and England [10]. Boston also had the highest median MME/day of 38 (IQR 20, 45). Both type of opioid and MME/day at first prescription have implications for future long-term opioid use, dependence, and unintentional overdoses reported frequently in the US, underscoring its importance [16–19].

The CDC recommendation for setting a recommended initial threshold of 50 MME/day or more following which to carefully assess individual benefits and risks came out in 2016, after the study period [12]. However, acknowledging this recommendation was not available at the time of the study; it is important to report prescribing patterns before this guideline was made available. High-risk initial opioid prescribing (≥50 MME/day) was observed in a fifth of Boston patients. A previous US study defining such behavior as ≥50 MME/day or a supply duration of >3 days estimated a monthly rate of 115,378 prescriptions per 15,897,673 patients in people on opioids [20]. Benzodiazepines were concurrently prescribed with opioids between 4% and 20% among the cohorts, most frequently in Quebec. Previous work has reported similar estimates of concomitant opioid and benzodiazepine overlap; however, proportions can vary considerably depending on the definition of concomitant use, country, and age group assessed [23,24]. Prescription of benzodiazepines with opioids has been associated with an increased risk of death from drug overdose, in a dose-dependent manner [25], and also resulted in a boxed warning by the FDA in 2016 against its use with opioids [26]. Concomitant gabapentinoid use was less frequent, up to 7% in Quebec, use of which with opioids has been associated with a 49% higher risk of opioid-related death in a previous Canadian population [27].

Overall, one of the highest rates of opioid prescribing was in Alberta, which is known to have substantially higher rates of opioid-related hospitalizations and deaths compared to other Canadian provinces [28,29]. Recent reports also document higher rates of opioid-related emergency department visits [30] and decreases in life expectancy in the province of Alberta [31]. Reasons for the high rates of opioid use in Alberta may be related to a higher prevalence of chronic pain problems than most other provinces [32] and will require further investigation.

The differences in baseline characteristics of patients between jurisdictions in themselves were not enough to explain the difference in age- and sex-standardized opioid prescribing rates (Table 1). When evaluated over time (2010 to 2015), we observed a higher proportion of older patients with more comorbidities (as measured by the Charlson comorbidity index) commenced on opioids within the Boston cohort, with more frequently reported pain diagnosis in later years (S2 Appendix). While the reasons for a global opioid crisis and increases in prescribing are likely to be multifactorial, reliance on prescription opioids for non-cancer pain management is likely to be a major contributor. Efforts to improve pain management have led to quadrupled rates of opioid prescribing [33], perhaps influenced by an aging population with more chronic pain and safety concerns with alternative analgesics including COX-II inhibitors [34,35], traditional NSAIDS [36], and paracetamol [37,38]. Country-specific drivers are likely to also have driven overprescribing. In the US, newer opioid formulations, aggressive marketing by pharmaceutical companies including direct advertising to patients that misrepresented harms, and changes in recommendations for treating chronic pain are all likely to have contributed [39]. Canada follows the US as the second highest per capita user of prescription opioids, with rates of high-dose opioid dispensing [40]. Variation in opioid coverage on public drug plans and differential marketing from pharmaceutical companies may all contribute.

Opioid use at initiation has been shown to affect downstream consequences such as long-term use [22] and unintentional overdoses, underscoring the importance of commencing on low potency and dose on first prescription. Recent studies beyond 2015 have demonstrated a decline or a plateauing of opioid prescribing across a number of jurisdictions, especially of high-dose use and of high-strength opioids such as oxycodone [22,41,42]. In all populations, the decline in such prescribing reflects national, regulatory, federal, statewide, and local efforts, especially as portrayals related to opioid-related harms become more prevalent via the media. In the US and Canada especially, a number of targeted opioid stewardship interventions that consist of a range of risk reduction strategies including prescription drug monitoring programs, dashboards for monitoring, clinical decision support, and audit and feedback tools have been successfully implemented [43–45]. However, despite falling opioid prescribing rates in the US, deaths involving synthetic opioids continued to increase in 2018 and accounted for two-thirds of opioid-involved deaths [4,46]. Additionally, the impact of COVID-19 on the opioid epidemic has led to a further escalation of opioid-related deaths within certain provinces in Canada [47]. More evidence will be required on the long-term consequences of the pandemic on opioid prescribing and related deaths.

### Strengths and limitations

To our knowledge, this is the first study comparing primary care opioid prescribing practices between several jurisdictions and countries highlighting important prescribing differences. Limitations include data from each jurisdiction were available at slightly different time windows during the 10-year observation period due to data availability. We had access to either prescribed or dispensed data, however, did not measure if opioids were administered by patients or issues around divergence. Some codeine containing preparations that were available over the counter without a prescription would be underrepresented in our cohort. Opioid prescribing systems also vary considerably between jurisdictions with a Prescription Drug Monitoring Programme database capturing statewide prior opioid use in Boston and weak opioid preparations that would be available over the counter in other countries than the Taiwanese cohort. The UK and US cohorts were created using all available data from certain technology systems, while the Quebec and Taiwan cohorts were population-based representative samples and Alberta included the entire population. While considerable efforts were made to harmonize the data from different sources, dissimilarities in coding and measurement may result in arbitrary differences between cohorts. For instance, while 69.9% of the Taiwan population had a pain diagnosis recorded at baseline, which is higher than other cohorts (Table 1), this is likely to be because of how pain is coded in this health system prior to prescription of opioids rather than due to a considerably higher prevalence of chronic pain.

## Conclusions

In one of the first multijurisdictional investigations of opioid drug utilization in non-cancer pain, to our knowledge, we demonstrated clear differences in patient characteristics, prescribing practices and rates per 1,000 population. While the opioid epidemic has affected the US more than other countries, opioid prescribing rates were higher within the same time period in certain Canadian provinces such as Alberta compared to Boston. While not always medically inappropriate, opioids with a high potential for abuse such as oxycodone should be restricted as first line only in exceptional cases for non-cancer pain where possible. Future work should focus on how different indications may lead to high-dose/potency use to inform targeted policy changes and how risks associated with individual drugs vary between jurisdictions in the context of country specific factors.

## Supporting information

S1 STROBE Checklist STROBE, STrengthening the Reporting of OBservational studies in Epidemiology. (DOC)

S1 Appendix Creating a new user cohort in all jurisdictions. Additional details about data sources. (DOCX)

S2 Appendix Baseline characteristics over time for each jurisdiction. (DOCX)

## Abbreviations

CDC: Centers for Disease Control and Prevention
COVID-19: Coronavirus Disease 2019
CPRD: Clinical Practice Research Datalink
EHR: electronic health record
MME: morphine milligram equivalent
NHI: National Health Insurance
