# Supplementary material for: Opioid prescribing among new users for non-cancer pain in the USA, Canada, UK, and Taiwan: A population-based cohort study
Source: PLoS Med. 2021 Nov 1;18(11):e1003829. doi: 10.1371/journal.pmed.1003829 (PMC8601614; doi:10.1371/journal.pmed.1003829)
Supplement: S1 Appendix — (DOCX) [file pmed.1003829.s002.docx]

**S1 Appendix:**

**Creating a New user cohort in all jurisdictions:**

First, we created a list of patients with prescription/dispensation of opioids between January 1, 2006 and January 1, 20016. For each patient and prescription combination, an indicator variable was created if the patient was ≥18 years of age on the date of prescription (1= patient ≥18 years, 0= patient is not ≥18 years). For each patient and prescription date combination a second indicator variable was created with a look back of 2 years from each prescription date, to determine if the patient was prescribed an opioid in the two years prior to prescription (1= incident user, 0=prevalent user). A worked example is shown below.

| Patient ID | Prescription date | Age on prescription date | ≥18 years indicator | New user indicator |
| --- | --- | --- | --- | --- |
| 1234 | 01 Feb 2013 | 16 | 0 | 1 |
| 1234 | 25 Apr 2015 | 18 | 1 | 1 |
| 5478 | 03 Apr 2007 | 45 | 1 | 0 |
| 5478 | 04 Jun 2008 | 46 | 1 | 0 |
| 5478 | 25 Oct 2009 | 47 | 1 | 1 |
| 5478 | 31 Dec 2011 | 49 | 1 | 0 |

For patient 1234, the index date would be for the prescription on the 25^th^ of April 2015. For patient 5478 the index date would be Oct 25^th^ 2009.

**Additional details about data sources:**

### *Canada: Quebec*

All medical services, hospitalizations (admission/discharge date, primary and secondary diagnoses), births and deaths were retrieved from the provincial health insurance agency that provides universal medical coverage for all Quebec residents. All dispensed prescriptions (prescriber, pharmacy, drug, dose, dispensing date, duration, refills) were available for the approximately 50% of provincial residents who are publicly insured.

*Canada: Alberta*

Alberta Health Services data include drugs dispensed from community pharmacies (prescriber, pharmacy, drug, dose, dispensing date, duration, refills), physician claims records (visit dates, ICD-9 diagnoses), inpatient and emergency records (admission and discharge dates, ICD-10 diagnoses and procedures) and deaths.

*United States: Boston, Massachusetts*

Partners HealthCare Research Patient Data Registry includes data from the Longitudinal Medical Record, an internally developed EHR for the participating clinics from Brigham and Women’s Hospital (BWH) and Massachusetts General Hospital (MGH). To ensure complete follow-up, patients were eligible if they were seen in one of 37 BWH- or MGH- affiliated primary care or diabetes clinics. Additionally, the Prescription Drug Monitoring Programme (PDMP) database provided state-wide individual-level data on prior opioid use based on dispensing information.

*United Kingdom*

CPRD Gold provides coverage for over 15 million patients in >700 general practices in the UK, representative of the national population. Patients were included if they had linkage to Hospital Episode Statistics data. The linked data includes information on all primary care interactions, including documented health problems, prescriptions, records of specialty referrals, hospitalizations and death.

*Taiwan*

The NHI’s research databases include a registry for beneficiaries (e.g. registrant’s age, sex, residence), an outpatient visit database (date and time of visit, ICD-9-CM codes of existing health problems, service provided), an inpatient visit database (date of hospitalization and discharge, ICD-9-CM codes of existing health problems, procedure codes and dates), and a pharmacy database (drug prescribed, date, duration, dosage, prescribing physician, dispensing pharmacy).
