# Supplementary material for: Opioid prescribing among new users for non-cancer pain in the USA, Canada, UK, and Taiwan: A population-based cohort study
Source: PLoS Med. 2021 Nov 1;18(11):e1003829. doi: 10.1371/journal.pmed.1003829 (PMC8601614; doi:10.1371/journal.pmed.1003829)

**S2 Appendix: Baseline characteristics over time for each jurisdiction**

| **Quebec** | **Year of the first opioid prescription** | | | | | | | | | | | | | | | | | |
| --- | --- | --- | --- | --- | --- | --- | --- | --- | --- | --- | --- | --- | --- | --- | --- | --- | --- | --- |
|  | **2006**  **(N=3,211)** | | **2007**  **(N=3,382)** | | **2008**  **(N=3,485)** | | **2009**  **(N=3,209)** | | **2010**  **(N=2,974)** | | **2011**  **(N=2,951)** | | **2012**  **(N=2,734)** | | **2013**  **(N=2,572)** | | **2014**  **(N=2,353)** | |
|  | N | % | N | % | N | % | N | % | N | % | N | % | N | % | N | % | N | % |
| **Age at first opioid** | 100 | 3.1 | 85 | 2.5 | 102 | 2.9 | 108 | 3.4 | 95 | 3.2 | 99 | 3.4 | 90 | 3.3 | 108 | 4.2 | 91 | 3.9 |
| 18 and 24 years |  |  |  |  |  |  |  |  |  |  |  |  |  |  |  |  |  |  |
| 25 and 34 years | 250 | 7.8 | 291 | 8.6 | 278 | 8.0 | 270 | 8.4 | 256 | 8.6 | 293 | 9.9 | 256 | 9.4 | 257 | 10.0 | 208 | 8.8 |
| 35 and 44 years | 416 | 13.0 | 410 | 12.1 | 372 | 10.7 | 346 | 10.8 | 354 | 11.9 | 316 | 10.7 | 292 | 10.7 | 296 | 11.5 | 246 | 10.5 |
| 45 and 54 years | 505 | 15.7 | 573 | 16.9 | 533 | 15.3 | 507 | 15.8 | 463 | 15.6 | 499 | 16.9 | 428 | 15.7 | 356 | 13.8 | 364 | 15.5 |
| 55 and 64 years | 547 | 17.0 | 577 | 17.1 | 567 | 16.3 | 537 | 16.7 | 467 | 15.7 | 479 | 16.2 | 419 | 15.3 | 459 | 17.9 | 387 | 16.5 |
| 65 and 74 years | 711 | 22.1 | 694 | 20.5 | 759 | 21.8 | 684 | 21.3 | 646 | 21.7 | 604 | 20.5 | 634 | 23.2 | 518 | 20.1 | 537 | 22.8 |
| ≥75 years | 682 | 21.2 | 752 | 22.2 | 874 | 25.1 | 757 | 23.6 | 693 | 23.3 | 661 | 22.4 | 615 | 22.5 | 578 | 22.5 | 520 | 22.1 |
| **Sex** | 954 | 29.7 | 1,048 | 31.0 | 1,067 | 30.6 | 991 | 30.9 | 904 | 30.4 | 949 | 32.2 | 896 | 32.8 | 866 | 33.7 | 790 | 33.6 |
| Male |  |  |  |  |  |  |  |  |  |  |  |  |  |  |  |  |  |  |
| Female | 2,257 | 70.3 | 2,334 | 69.0 | 2,418 | 69.4 | 2,218 | 69.1 | 2,070 | 69.6 | 2,002 | 67.8 | 1,838 | 67.2 | 1,706 | 66.3 | 1,563 | 66.4 |
| **Charlson Comorbidity index** | 1,774 | 55.2 | 1,868 | 55.2 | 1,844 | 52.9 | 1,728 | 53.8 | 1,575 | 53.0 | 1,658 | 56.2 | 1,527 | 55.9 | 1,456 | 56.6 | 1,336 | 56.8 |
| Very low score (0) |  |  |  |  |  |  |  |  |  |  |  |  |  |  |  |  |  |  |
| Low score (1) | 918 | 28.6 | 905 | 26.8 | 952 | 27.3 | 892 | 27.8 | 833 | 28.0 | 777 | 26.3 | 692 | 25.3 | 664 | 25.8 | 597 | 25.4 |
| Medium score (2-3) | 412 | 12.8 | 482 | 14.3 | 541 | 15.5 | 443 | 13.8 | 426 | 14.3 | 372 | 12.6 | 377 | 13.8 | 335 | 13.0 | 289 | 12.3 |
| High score (≥4) | 107 | 3.3 | 127 | 3.8 | 148 | 4.3 | 146 | 4.6 | 140 | 4.7 | 144 | 4.9 | 138 | 5.1 | 117 | 4.6 | 131 | 5.6 |
| **Comorbidity in 2 years before 1^st^ opioid** | |  |  |  |  |  |  |  |  |  |  |  |  |  |  |  |  |  |
| Depression | 558 | 17.4 | 615 | 18.2 | 656 | 18.8 | 638 | 19.9 | 570 | 19.2 | 609 | 20.6 | 626 | 22.9 | 581 | 22.6 | 551 | 23.4 |
| Substance abuse | 243 | 7.6 | 222 | 6.6 | 220 | 6.3 | 227 | 7.1 | 191 | 6.4 | 194 | 6.6 | 192 | 7.0 | 164 | 6.4 | 154 | 6.5 |
| Pain | 994 | 31.0 | 1,020 | 30.2 | 974 | 28.0 | 900 | 28.1 | 778 | 26.2 | 796 | 27.0 | 693 | 25.4 | 665 | 25.9 | 566 | 24.1 |
| **Concurrent drug use on the date 1^st^ opioid** | |  |  |  |  |  |  |  |  |  |  |  |  |  |  |  |  |  |
| Gabapentinoids | 105 | 3.3 | 152 | 4.5 | 237 | 6.8 | 243 | 7.6 | 235 | 7.9 | 235 | 8.0 | 266 | 9.7 | 249 | 9.7 | 253 | 10.8 |
| Benzodiazepines | 671 | 21.0 | 697 | 20.6 | 768 | 22.0 | 697 | 21.7 | 624 | 21.0 | 555 | 18.8 | 528 | 19.3 | 451 | 17.5 | 369 | 15.7 |
| Antipsychotics | 145 | 4.5 | 221 | 6.5 | 234 | 6.7 | 233 | 7.3 | 265 | 8.9 | 243 | 8.2 | 326 | 11.9 | 304 | 11.8 | 297 | 12.6 |
| Antidepressants | 770 | 24.0 | 912 | 27.0 | 1,104 | 31.7 | 1,182 | 36.8 | 1,130 | 38.0 | 1,137 | 38.5 | 1,176 | 43.0 | 1,153 | 44.8 | 1,126 | 47.9 |

| **Taiwan** | **Year of the first opioid prescription** | | | | | | | |
| --- | --- | --- | --- | --- | --- | --- | --- | --- |
|  | **2009**  **(N=8,109)** | | **2010**  **(N=9,576)** | | **2011**  **(N=10,240)** | | **2012**  **(N=10,329)** | |
|  | N | % | N | % | N | % | N | % |
| **Age at first opioid** | 432 | 5.3 | 528 | 5.5 | 570 | 5.6 | 580 | 5.6 |
| 18 and 24 years |  |  |  |  |  |  |  |  |
| 25 and 34 years | 924 | 11.4 | 1,124 | 11.7 | 1,196 | 11.7 | 1,189 | 11.5 |
| 35 and 44 years | 1,116 | 13.8 | 1,313 | 13.7 | 1,422 | 13.9 | 1,426 | 13.8 |
| 45 and 54 years | 1,637 | 20.2 | 1,909 | 19.9 | 1,925 | 18.8 | 1,866 | 18.1 |
| 55 and 64 years | 1,509 | 18.6 | 1,810 | 18.9 | 2,009 | 19.6 | 2,105 | 20.4 |
| 65 and 74 years | 1,315 | 16.2 | 1,493 | 15.6 | 1,529 | 14.9 | 1,468 | 14.2 |
| ≥75 years | 1,176 | 14.5 | 1,399 | 14.6 | 1,589 | 15.5 | 1,695 | 16.4 |
| **Sex** | 3,458 | 42.6 | 4,264 | 44.5 | 4,521 | 44.2 | 4,721 | 45.7 |
| Male |  |  |  |  |  |  |  |  |
| Female | 4,651 | 57.4 | 5,312 | 55.5 | 5,719 | 55.9 | 5,608 | 54.3 |
| **Charlson Comorbidity index** | 3,707 | 45.7 | 4,520 | 47.2 | 4,828 | 47.2 | 5,039 | 48.8 |
| Very low score (0) |  |  |  |  |  |  |  |  |
| Low score (1) | 2,331 | 28.8 | 2,707 | 28.3 | 2,816 | 27.5 | 2,860 | 27.7 |
| Medium score (2-3) | 1,650 | 20.3 | 1,874 | 19.6 | 2,073 | 20.2 | 1,960 | 19.0 |
| High score (≥4) | 421 | 5.2 | 475 | 5.0 | 523 | 5.1 | 470 | 4.6 |
| **Comorbidity in 2 years before 1^st^ opioid** | |  |  |  |  |  |  |  |
| Depression | 162 | 2.0 | 185 | 1.9 | 234 | 2.3 | 220 | 2.1 |
| Substance abuse | 54 | 0.7 | 79 | 0.8 | 80 | 0.8 | 77 | 0.7 |
| Pain | 5,829 | 71.9 | 6,794 | 71.0. | 7,100 | 69.4 | 7,000 | 67.8 |
| **Concurrent drug use on the date 1^st^ opioid** | |  |  |  |  |  |  |  |
| Gabapentinoids | 159 | 2.0 | 184 | 1.9 | 193 | 1.9 | 219 | 2.1 |
| Benzodiazepines | 1,499 | 18.5 | 1,712 | 17.9 | 1,856 | 18.1 | 1,913 | 18.5 |
| Antipsychotics | 234 | 2.9 | 300 | 3.1 | 333 | 3.3 | 365 | 3.5 |
| Antidepressants | 567 | 7.0 | 646 | 6.8 | 712 | 7.0 | 680 | 6.6 |

| **UK** | **Year of the first opioid prescription** | | | | | | | | | | | | | | | | | | | |
| --- | --- | --- | --- | --- | --- | --- | --- | --- | --- | --- | --- | --- | --- | --- | --- | --- | --- | --- | --- | --- |
|  | **2006 (N=126,835)** | | **2007 (N=137,239)** | | **2008 (N=133,683)** | | **2009 (N=124,370)** | | **2010 (N=112,856)** | | **2011 (N=100,799)** | | **2012 (N=92,074)** | | **2013**  **(N=78,028)** | | **2014**  **(N=62,164)** | | **2015**  **(N=44,891)** | |
|  | N | % | N | % | N | % | N | % | N | % | N | % | N | % | N | % | N | % | N | % |
| **Age at first opioid** | 8,672 | 6.8 | 9,252 | 6.7 | 9,197 | 6.9 | 9,080 | 7.3 | 8,452 | 7.5 | 8,127 | 8.1 | 7,881 | 8.6 | 6,894 | 8.8 | 5,607 | 9.0 | 4,143 | 9.2 |
| 18 and 24 years |  |  |  |  |  |  |  |  |  |  |  |  |  |  |  |  |  |  |  |  |
| 25 and 34 years | 17,114 | 13.5 | 18,039 | 13.1 | 17,605 | 13.2 | 16,953 | 13.6 | 15,730 | 13.9 | 14,334 | 14.2 | 13,892 | 15.1 | 12,167 | 15.6 | 9,760 | 15.7 | 7,017 | 15.6 |
| 35 and 44 years | 22,701 | 17.9 | 24,464 | 17.8 | 23,463 | 17.6 | 21,705 | 17..4 | 19,503 | 17.3 | 17,140 | 17.0 | 15,639 | 17.0 | 12,770 | 16.4 | 10,186 | 16.4 | 7,053 | 15.7 |
| 45 and 54 years | 20,684 | 16.3 | 22,792 | 16.6 | 22,249 | 16.6 | 21,158 | 17.0 | 19,801 | 17.6 | 17,615 | 17.5 | 16,500 | 17.9 | 13,782 | 17.7 | 10,792 | 17.4 | 7,894 | 17.6 |
| 55 and 64 years | 21,147 | 16.7 | 22,960 | 16.7 | 22,476 | 16.8 | 20,405 | 16.4 | 18,252 | 16.2 | 16,007 | 15.9 | 13,881 | 15.1 | 11,635 | 14.9 | 9,149 | 14.7 | 6,543 | 14.6 |
| 65 and 74 years | 16,963 | 13.4 | 18,435 | 13.4 | 18,046 | 13.5 | 16,553 | 13.3 | 14,786 | 13.1 | 13,104 | 13.0 | 11,703 | 12.7 | 9,908 | 12.7 | 8,000 | 12.9 | 5,887 | 13.1 |
| ≥75 years | 19,554 | 15.4 | 21,297 | 15.5 | 20,647 | 15.4 | 18,516 | 14.9 | 16,332 | 14.5 | 14,472 | 14.4 | 12,578 | 13.7 | 10,872 | 13.9 | 8,670 | 14.0 | 6,354 | 14.2 |
| **Sex** | 52,521 | 41.4 | 56,511 | 41.2 | 55,214 | 41.3 | 52,490 | 42.2 | 48,066 | 42.6 | 43,280 | 42.9 | 39,516 | 42.9 | 33,598 | 43.1 | 27,036 | 43.5 | 19,401 | 43.2 |
| Male |  |  |  |  |  |  |  |  |  |  |  |  |  |  |  |  |  |  |  |  |
| Female | 74,314 | 58.6 | 80,728 | 58.8 | 78,469 | 58.7 | 71,880 | 57.8 | 64,790 | 57.4 | 57,519 | 57.1 | 52,558 | 57.1 | 44,430 | 56.9 | 35,128 | 56.5 | 25,490 | 56.8 |
| **Charlson Comorbidity index** | |  |  |  |  |  |  |  |  |  |  |  |  |  |  |  |  |  |  |  |
| Very low score (0) | 86,304 | 68.0 | 89,916 | 65.5 | 85,858 | 64.2 | 79,828 | 64.2 | 72,317 | 64.1 | 64,115 | 63.6 | 59,038 | 64.1 | 49,409 | 63.3 | 39,339 | 63.3 | 27,815 | 62.0 |
| Low score (1) | 26,380 | 20.8 | 27,948 | 20.4 | 27,158 | 20.3 | 25,002 | 20.1 | 22,643 | 20.1 | 20,408 | 20.3 | 18,459 | 20.1 | 16,120 | 20.6 | 12,827 | 20.6 | 9,477 | 21.1 |
| Medium score (2-3) | 10,871 | 8.6 | 14,282 | 10.4 | 15,009 | 11.2 | 14,097 | 11.3 | 12,865 | 11.4 | 11,450 | 11.4 | 10,177 | 11.1 | 8,648 | 11.1 | 6,857 | 11.0 | 5,125 | 11.4 |
| High score (≥4) | 3,280 | 2.6 | 5,093 | 3.7 | 5,658 | 4.2 | 5,443 | 4.4 | 5,031 | 4.5 | 4,826 | 4.8 | 4,400 | 4.8 | 3,851 | 4.9 | 3,141 | 5.1 | 2,474 | 5.5 |
| **Comorbidity in 2 years before 1^st^ opioid** | | |  |  |  |  |  |  |  |  |  |  |  |  |  |  |  |  |  |  |
| Depression | 12,187 | 9.6 | 12,650 | 9.2 | 11,643 | 8.7 | 10,206 | 8.2 | 9,217 | 8.2 | 8,578 | 8.5 | 8,042 | 8.7 | 6,894 | 8.8 | 5,205 | 8.4 | 3,864 | 8.6 |
| Substance abuse | 2,362 | 1.9 | 2,593 | 1.9 | 2,513 | 1.9 | 2,546 | 2.0 | 2,560 | 2.3 | 3,683 | 3.6 | 5,179 | 5.6 | 5,359 | 6.9 | 4,421 | 7.1 | 3,078 | 6.9 |
| Pain | 38,487 | 30.4 | 41,177 | 30.0 | 40,370 | 30.2 | 37,047 | 29.8 | 33,500 | 29.7 | 29,642 | 29.4 | 26,546 | 28.8 | 22,002 | 28.2 | 17,435 | 28.1 | 12,456 | 27.8 |
| **Concurrent drug use on the date 1^st^ opioid** | | |  |  |  |  |  |  |  |  |  |  |  |  |  |  |  |  |  |  |
| Gabapentinoids | 755 | 0.6 | 1,024 | 0.8 | 1,137 | 0.9 | 1,163 | 0.9 | 1,318 | 1.2 | 1,357 | 1.4 | 1,516 | 1.7 | 1,518 | 2.0 | 1,437 | 2.3 | 1,222 | 2.7 |
| Benzodiazepines | 8,717 | 6.9 | 9,481 | 6.9 | 9,286 | 7.0 | 8,722 | 7.0 | 7,896 | 7.0 | 7,188 | 7.1 | 6,400 | 7.0 | 5,639 | 7.2 | 4,443 | 7.2 | 3,269 | 7.3 |
| Antipsychotics | 2,995 | 2.4 | 3,101 | 2.3 | 3,192 | 2.4 | 2,829 | 2.3 | 2,384 | 2.1 | 2,151 | 2.1 | 1,793 | 2.0 | 1,689 | 2.2 | 1,383 | 2.2 | 962 | 2.1 |
| Antidepressants | 8,449 | 6.7 | 9,244 | 6.7 | 9,530 | 7.1 | 8,651 | 7.0 | 7,928 | 7.0 | 7,331 | 7.3 | 6,638 | 7.2 | 6,040 | 7.7 | 5,044 | 8.1 | 3,767 | 8.4 |

| **Boston** | **Year of the first opioid prescription** | | | | | | | | | | | | | |  |
| --- | --- | --- | --- | --- | --- | --- | --- | --- | --- | --- | --- | --- | --- | --- | --- |
|  | **2010**  **(N=4,269)** | | **2011**  **(N=5,274)** | | **2012**  **(N=6,384)** | | **2013**  **(N=7,652)** | | **2014 (**  **N=9,432)** | | **2015**  **(N=8,422)** | | **2016**  **(N=3,257)** | |  |
|  | N | % | N | % | N | % | N | % | N | % | N | % | N | % |  |
| **Age at first opioid** | 304 | 7.1 | 364 | 6.9 | 443 | 6.9 | 385 | 5.0 | 406 | 4.3 | 231 | 2.7 | 191 | 5.9 |  |
| 18 and 24 years |  |  |  |  |  |  |  |  |  |  |  |  |  |  |  |
| 25 and 34 years | 827 | 19.4 | 969 | 18.4 | 1,172 | 18.4 | 1,400 | 18.3 | 1,674 | 17.8 | 1,160 | 13.8 | 579 | 17.8 |  |
| 35 and 44 years | 969 | 22.7 | 1,195 | 22.7 | 1,374 | 21.5 | 1,566 | 20.5 | 1,922 | 20.4 | 1,460 | 17.3 | 586 | 18.0 |  |
| 45 and 54 years | 928 | 21.7 | 1,124 | 21.3 | 1,346 | 21.1 | 1,598 | 20.9 | 1,970 | 20.9 | 1,785 | 21.2 | 620 | 19.0 |  |
| 55 and 64 years | 693 | 16.2 | 892 | 16.9 | 1,069 | 16.7 | 1,438 | 18.8 | 1,792 | 19.0 | 1,894 | 22.5 | 632 | 19.4 |  |
| 65 and 74 years | 345 | 8.1 | 451 | 8.6 | 635 | 9.9 | 776 | 10.1 | 1,096 | 11.6 | 1,211 | 14.4 | 416 | 12.8 |  |
| ≥75 years | 203 | 4.7 | 279 | 5.4 | 345 | 5.4 | 489 | 6.4 | 572 | 6.1 | 681 | 8.1 | 233 | 7.2 |  |
| **Sex** | 1,408 | 32.9 | 1,774 | 33.6 | 2,116 | 33.2 | 2,445 | 32.0 | 2,969 | 31.4 | 2,698 | 32.0 | 1,159 | 35.6 |  |
| Male |  |  |  |  |  |  |  |  |  |  |  |  |  |  |  |
| Female | 2,861 | 67.0 | 3,500 | 66.4 | 4,268 | 66.8 | 5,207 | 68.1 | 6,463 | 68.5 | 5,724 | 68.0 | 2,098 | 64.4 |  |
| **Charlson Comorbidity index** | 3,128 | 73.3 | 3,808 | 72.2 | 4,549 | 71.3 | 5,281 | 69.0 | 6,260 | 66.4 | 5,321 | 63.2 | 1,939 | 59.5 |  |
| Very low score (0) |  |  |  |  |  |  |  |  |  |  |  |  |  |  |  |
| Low score (1) | 657 | 15.4 | 857 | 16.3 | 998 | 15.6 | 1,339 | 17.5 | 1,789 | 19.0 | 1,598 | 19.0 | 701 | 21.5 |  |
| Medium score (2-3) | 333 | 7.8 | 443 | 8.4 | 591 | 9.3 | 743 | 9.7 | 974 | 10.3 | 961 | 11.4 | 382 | 11.7 |  |
| High score (≥4) | 151 | 3.5 | 166 | 3.2 | 246 | 3.8 | 289 | 3.8 | 409 | 4.3 | 542 | 6.4 | 235 | 7.2 |  |
| **Comorbidity in 2 years before 1^st^ opioid** | |  |  |  |  |  |  |  |  |  |  |  |  |  |  |
| Depression | 364 | 8.5 | 412 | 7.8 | 559 | 8.8 | 656 | 8.6 | 799 | 8.5 | 846 | 10.1 | 259 | 8.0 |  |
| Substance abuse | 174 | 4.1 | 165 | 3.1 | 198 | 3.1 | 235 | 3.1 | 258 | 2.7 | 293 | 3.5 | 47 | 1.4 |  |
| Pain | 864 | 20.2 | 1,075 | 20.4 | 1,362 | 21.3 | 1,714 | 22.4 | 2,208 | 23.4 | 2,238 | 26.6 | 346 | 10.6 |  |
| **Concurrent drug use on the date 1^st^ opioid** | |  |  |  |  |  |  |  |  |  |  |  |  |  |  |
| Gabapentinoids | 71 | 1.7 | 102 | 1.9 | 137 | 2.2 | 183 | 2.4 | 271 | 2.9 | 465 | 5.5 | 188 | 5.8 |  |
| Benzodiazepines | 247 | 5.8 | 331 | 6.3 | 407 | 6.4 | 497 | 6.5 | 616 | 6.5 | 836 | 9.9 | 277 | 8.5 |  |
| Antipsychotics | 57 | 1.3 | 80 | 1.5 | 84 | 1.3 | 92 | 1.2 | 124 | 1.3 | 160 | 1.9 | 53 | 1.6 |  |
| Antidepressants | 124 | 2.9 | 154 | 2.9 | 216 | 3.4 | 276 | 3.6 | 365 | 3.9 | 713 | 8.5 | 216 | 6.6 |  |


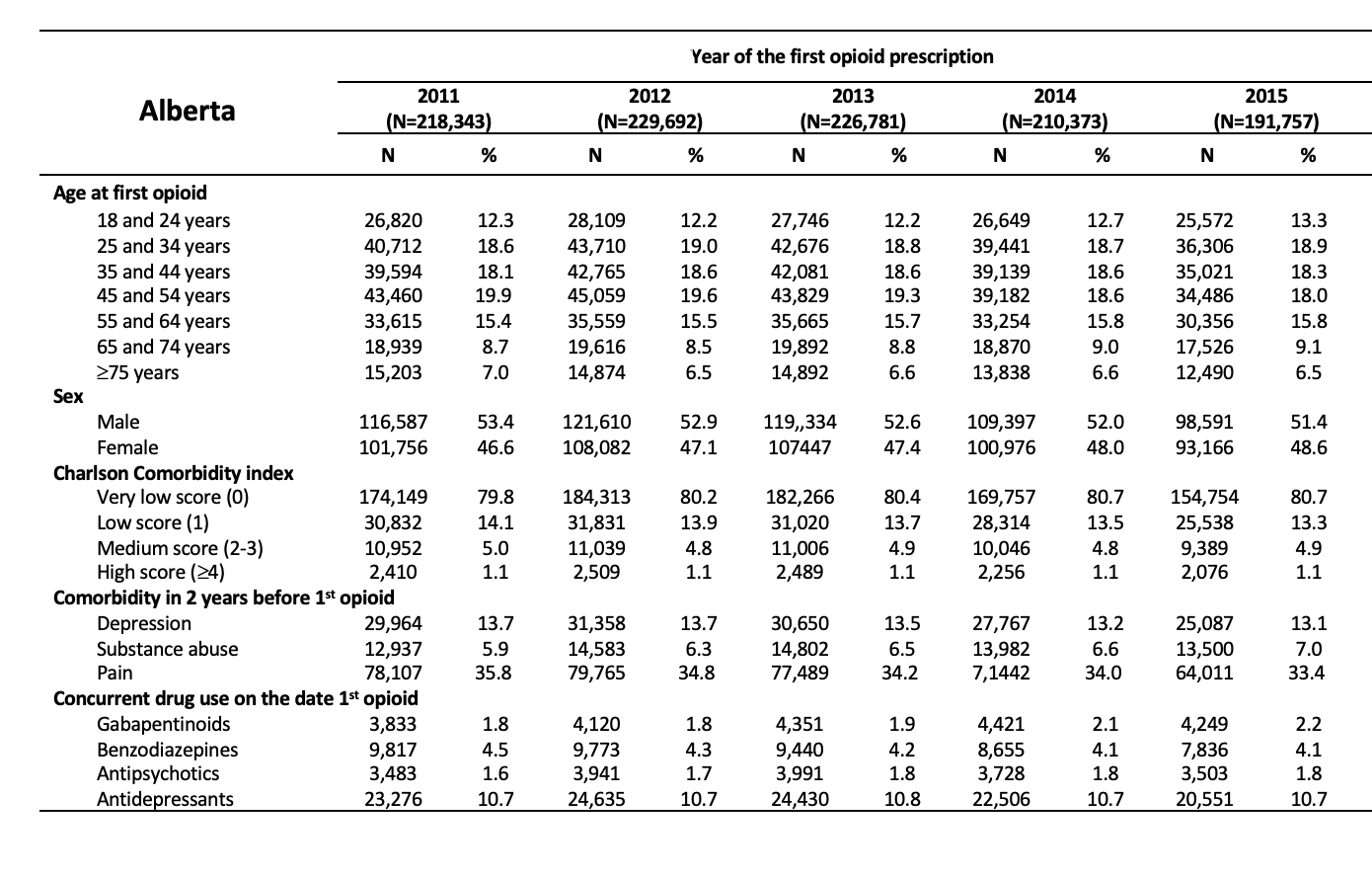

Supplement: S2 Appendix — (DOCX) [file pmed.1003829.s003.docx]
